# Relationship between albumin and rheumatoid arthritis: Evidence from NHANES and Mendelian randomization

**DOI:** 10.1097/MD.0000000000039776

**Published:** 2024-10-11

**Authors:** Ke Liu, Le Zhang, Haoming Zhao, Zuyu Tang, Sheng Hua, Yixiao Xiong, Ziming Zhang, Liang Ou, Jianjun Kuang

**Affiliations:** a Hunan University of Chinese Medicine, Changsha, Hunan, China; b Hunan Academy of Chinese Medicine, Changsha, Hunan, China; c The Affiliated Hospital Of Hunan Academy Of Traditional Chinese Medicine, Changsha, Hunan, China.

**Keywords:** albumin, Mendelian randomization, NHANES, rheumatoid arthritis, serum proteins

## Abstract

With the rising incidence of rheumatoid arthritis (RA) and the increasing percentage of serum RF negativity, more and more accurate methods are urgently needed for the early diagnosis and prevention of RA, among which serum albumin (ALB) is closely related to the development of RA, and it is expected to become a new auxiliary diagnostic means, but its relationship with RA is not clear, so the present study aimed to investigate the Causal relationship. In this study, we used a generalized linear model and smoothed curve fitting to comprehensively evaluate the relationship between ALB and RA through the data of ALB and RA in the NHANES database, in addition, we further used inverse variance weighted (IVW) in Mendelian randomization (MR) in conjunction with the other 4 methods to further validate and clarify the causal relationship. The results were also examined for heterogeneity and horizontal pleiotropy to assess whether the results were robust. Finally, we used Bayesian co-localization analysis to clarify that ALB and RA share common genetic loci. In the observational study, after correction for multiple confounders, ALB remained more significantly negatively associated with RA (OR = 0.66, [95% CI = 0.51–0.86], *P* = .003), and subgroup analyses showed significant negative associations in both men and women (men: OR = 0.67, [95% CI = 0.46–0.99], *P* = .046; females: OR = 0.66, [95% CI = 0.44–1.00], *P* = .049). In further MR analysis, IVW: ALB on RA, OR = 0.70 [95% 0.52–0.93], *P* = .013; RA on ALB, OR = 0.95 [95% CI = 0.93–0.98], *P* < .001. The results of the MR analyses were in agreement with those of NHANES, which did not share a common genetic locus in co-localization analysis. There is a significant relationship between ALB and RA, and the reduction of ALB may be one of the risk factors for RA, as well as one of the outcomes in the development of RA.

## 1. Introduction

Rheumatoid arthritis (RA) is a chronic systemic immune disease that erodes symmetrically and causes inflammation of the joints, with pannus and synovitis as its main pathologic features, morning stiffness, swelling, and deformity of the joints, and bone destruction as its clinical manifestations.^[1]^ Global epidemiology shows that from 1990 to 2017, the global prevalence of the disease has increased by 7.4%, the incidence of the disease has increased by 8.2%, and the number of people with disabilities has risen year by year.^[2]^ Currently, the disease is mainly improved and slowed down by conventional antirheumatic drugs, biologics, and targeted drugs.^[3]^ Although it is found that genetics, gender, and various environmental factors may be related to the development of RA,^[4]^ there are still some pathological mechanisms that have not been fully clarified. RA is often diagnosed by serum testing combined with clinical symptoms, but the incidence of serum RF-positive RA is gradually decreasing, while the incidence of serum RF-negative RA is significantly increasing.^[5,6]^ Therefore, it is of great significance to explore the methods of early diagnosis and prevention of RA.

Biomarkers can be used for the diagnosis of RA patients, assessment of disease changes, and response to treatment,^[7]^ of which serum proteins are susceptible to disease and readily available, and have a role in the identification of RA.^[8]^ Serum proteins are easily affected by the disease and are readily available. Serum proteins, including ALB, are the most important proteins in human plasma and play an important role in maintaining nutrition and osmolality. In addition, several studies have shown an association between ALB and RA disease activity.^[9–12]^ It presents lower levels during the acute inflammatory response.^[13]^ In addition, fibroblasts play a key role in immune regulation through the production of various proteins, including ALB, in both healthy and diseased states. They are involved in lymphocyte migration, complement activation, inflammation, acute phase response and immune regulation. These molecules have an important role in autoinflammatory diseases such as RA.^[14]^

Although these small amounts of evidence above suggest that there is some association between certain serum ALB and RA, there is a lack of evidence supported by large data and large sample sizes.^[15]^ so we conducted this study to obtain a higher level of evidence to determine their relationship.

## 2. Methods

### 2.1. Transect studies

#### 2.1.1. Study design and participants

NHANES is a cross-sectional, population-based survey conducted by the Centers for Disease Control and Prevention (CDC) to assess the health and nutritional status of adults and children in the U.S. The NHANES survey provides a rich, realistic, and representative set of data for Clinical Research Health and Nutrition Data. We included NHANES data from 1999 to 2016, totaling 9 cycles, in our study. Participants with complete information on RA, ALB and covariates after screening were included in the study.

#### 2.1.2. NHANES ALB and RA Assessment

For participants, ALB was assessed by Standard Biochemistry Profile from Laboratory Data. RA was assessed by arthritis-related questions from the Questionnaire. Participants were asked 2 questions related to RA: has a doctor or other health professional ever told {you/SP} that {you/s/he}...had arthritis?; which type of arthritis was it? If the participant answered “yes” to the first question and “RA” to the second question, the participant was determined to be an individual with RA, otherwise a non-RA individual.

#### 2.1.3. Other covariates used in NHANES

To control for potential confounders, we included the following covariates: age,^[16]^ gender,^[2]^ ethnicity,^[2]^ education,^[17]^ income,^[2]^ smoke,^[18]^ alcohol,^[19,20]^ BMI,^[21]^ hypertension,^[22]^ high cholesterol,^[22]^ blood glucose.^[23]^ These confounders were chosen to consider possible associations with the prevalence of RA.

#### 2.1.4. NHANES analysis

We first conducted an NHANES analysis using 3 generalized linear models were designed to assess the relationship between ALB and RA. Model 1 was unadjusted (including gender); model 2 was adjusted for gender, age, race, education, and income; and model 3 added adjustments for smoking, alcohol consumption, body mass index, hypertension, hyperlipidemia, and serum glucose to model 2. After that, we use the smoothed curve fitting based on the generalized additive model to observe the trend and the presence or absence of a threshold between the 2, and further analyze the trend change before and after the threshold point, and judge whether there is a nonlinear relationship based on the log-likelihood ratio test. Considering the differences in RA by gender, we analyzed the subgroups by gender in the above studies. In addition, NHANES uses complex multistage probability sampling, and this study corrected for multistage, stratification, over-sampling, and weighting in the generalized linear model and population description to ensure the accuracy of the results.

### 2.2. Mendelian randomization

#### 2.2.1. Research design

Mendelian randomization (MR) is a statistical method for estimating the causal effect of exposure factors on outcome variables. Similar to a randomized controlled trial and because genes follow the principle of random assignment, the results are not subject to the confounding factors and reverse causal associations found in traditional epidemiological studies.MR determines the relationship between exposure and outcome through the combination of the exposure and the outcome’s single nucleotide polymorphism (SNP). SNPs as working variables need to meet 3 major assumptions: the assumption of association, where the SNP needs to be strongly associated with the exposure factor; the assumption of exclusivity, where this SNP can only affect the outcome by influencing the exposure and not through other pathways or modalities; and the assumption of independence, where this SNP can only affect the exposure and not directly the outcome. We obtained genome-wide association study (GWAS) summary statistics related to this study through the IEU OpenGWAS project (https://gwas.mrcieu.ac.uk/).

#### 2.2.2. MR analysis

We subjected ALB and RA to MR analysis to further validate their correlation and causation. We used the RA dataset (GWAS ID, ebi-a-GCST90018910) summarized from the study of Saori Sakaue et al^[24]^ which contains 417,256 sample sizes. the serum ALB dataset (GWAS ID, ebi-a-GCST90013990) summarized by Mbatchou J et al^[25]^ contains a total of 357,968 sample sizes. To obtain SNP values strongly correlated with exposure, the *P*-value between SNP and exposure was set at a significant level < 5e-08, and to exclude SNPs with linkage disequilibrium, r^2^ < 0.001 and clumping distance = 10,000 kb were set. Instrumental variables that do not strongly correlate with exposure factors or can only explain a small portion of the phenotypic variance were removed, and finally all *F*-test values were required to be >10, and we removed SNPs associated with confounding factors with the help of R. After that, MR analyses were performed.

In this study, inverse variance weighted (IVW), which has the strongest causal detection capability, was used as the main research method, however, the IVW method requires that genetic variation can only affect the results through exposure, so we used a more stringent approach, and all covariates of model 3 in the NHANES study were used as confounders in order to minimize the bias generated by confounders, and the removal of confounders was carried out with the help of the R package, and the confounders The keywords were set as age, income, BMI, body mass index, obesity, fat, glucose, diabetes, ethnicity, race, education, smoke, alcohol, hypertension, blood pressure, cholesterol, triglyceride, and reverse MR set the confounding factor keywords to liver, cancer, and nutrition. The causality was also determined by combining the other 4 MR methods (MR-Egger, weighted median, Simple mode, Weighted mode), and if the results of the 5 methods were similar and the IVW method was significant, a causal relationship was considered. In addition, we used Cochran *Q* test to assess heterogeneity, Egger intercept to assess horizontal pleiotropy, and MR-PRESSO to detect abnormal SNPs (outliers), and exclude outlier SNPs to obtain estimates closer to the true values. Finally, the “MR-PRESSO distortion test” was utilized to examine whether there is a difference between the pre-corrected and post-corrected results. leave-one-out was used to exclude each SNP 1 by 1 and the remaining SNPs were recalculated to see whether the results were significantly affected by a particular SNP. We plotted the results as a scatterplot, where each point corresponds to an SNP, showing the association between that genetic variant and exposure and outcome. Lines of different colors indicate fitting by different methods, showing the association between exposure and outcome predicted by all SNPs. Forest plots show the effect size of each SNP and its 95% confidence interval. Funnel plots are used to detect heterogeneity among genetic variants. If the funnel plot shows a symmetrical shape, this usually means that there is no significant heterogeneity, i.e., there is no systematic bias between the study effect and its accuracy. All exposures and outcomes were analyzed by bidirectional MR to clarify the presence of bidirectional causal effects.

#### 2.2.3. Bayesian co-location analysis

Quantitative trait loci (QTL) refer to loci on the genome, regions of genes that have some quantitative effect on specific traits. Among them, protein QTL (pQTL) are the closest and most stable QTL to the phenotype under study due to their ability to specifically regulate the level of protein expression. pQTL data for serum albumin were obtained from the deCODE database, and Bayesian co-localization analyses were used to determine whether serum albumin and RA were driven by the same region of the same causal variant locus. There were 4 hypotheses for the Bayesian co-localization analysis. When the posterior probability of hypothesis 4 (PH4) is greater than or equal to 0.80 is considered to be strong evidence of co-localization, this analysis strengthens the evidence of association between the 2 phenotypes and helps us to understand the relationship between the different phenotypes.

All NHANES statistical analyses were performed using EmpowerStats software. MR analyses were performed with the help of TwoSampleMR,^[26]^ MR-PRESSO,^[27]^ LdlinkR,^[28,29]^ coloc,^[30]^ dplyr, grid, and Forestploter Packages on the R 4.3.3 platform.

## 3. Results

### 3.1. Results of NHANES analysis

#### 3.1.1. Epidemiological observational analysis

**Figure 1.**
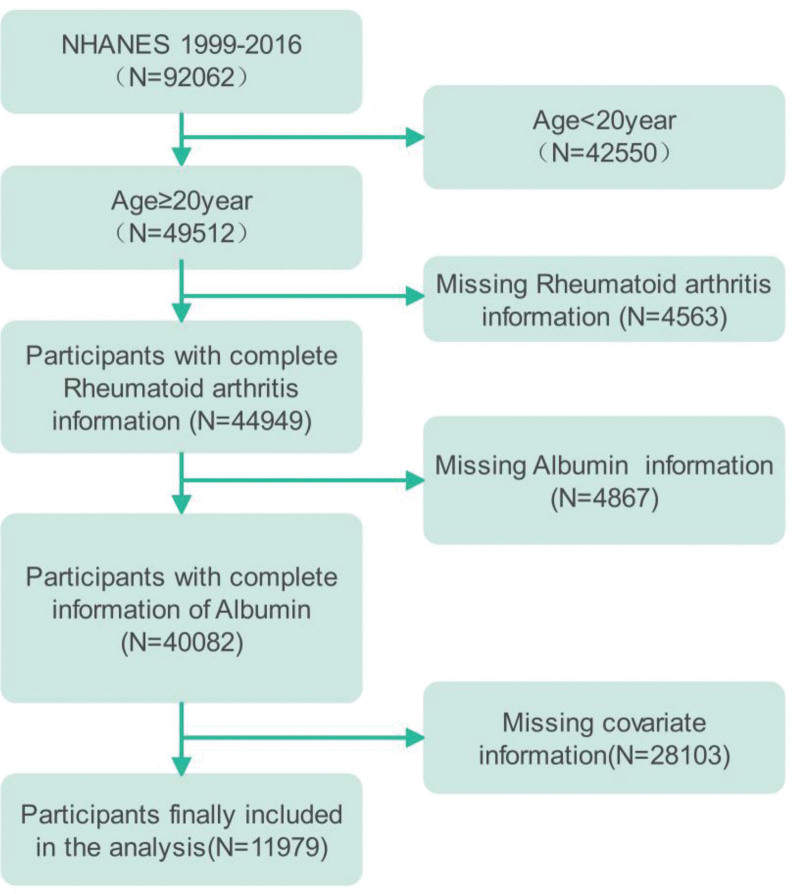
NHANES inclusion, exclusion process.

In the 1999 to 2016 study cycle, after screening the final 11,979 participants were included in the research study, of which there were 983 patients with RA, accounting for 8.21% of the total number of the study, and the inclusion and exclusion process is shown in Figure 1. The final inclusion population was analyzed epidemiologically under the consideration of the weights, of which 58.3% of the males and 41.7% of the females were analyzed in terms of the subgroups by gender under the analysis of weights, most of the male RA patients were elderly, low-income, smoking, drinking, hypertension, hyperlipidemia, hyperglycemia, and there were differences between different education levels and races, and there were no significant differences in body weight and smoking history between RA and non-RA. The population distribution trends of female RA patients were generally similar to those of male patients, but there were also differences in weight and smoking history between female RA and non-RA. Therefore these could be potential influencing factors for RA, and the results are shown in Table 1.

**Table 1:** Demographic Characteristics, Associated Diseases, Weighted, of RA or Non-RA Participants from the 1999 to 2016 cycle of NHANES.

Characteristics	Total	Male			Female		
		Non-RA	RA	*P*-value	Non-RA	RA	*P*-value
Age (yr)	50.4 (50.0,50.8)	50.2 (49.7,50.7)	58.4 (56.6,60.2)	<.0001	49.5 (49.0,50.1)	56.8 (55.4,58.1)	<.0001
Income	3.0 (3.0,3.1)	3.1 (3.1,3.2)	2.6 (2.4,2.9)	<.0001	3.0 (2.9,3.1)	2.3 (2.1,2.5)	<.0001
BMI (kg/m^2^)	28.8 (28.7,29.0)	28.8 (28.6,28.9)	29.3 (28.6,30.1)	.1019	28.8 (28.5,29.0)	30.6 (29.6,31.5)	.0004
Blood glucose (mmol/L)	5.5 (5.5,5.6)	5.7 (5.6,5.7)	5.9 (5.7,6.0)	.0421	5.4 (5.3,5.4)	5.6 (5.5,5.8)	.0015
SAL (g/dL)	4.3 (4.3,4.3)	4.4 (4.4,4.4)	4.3 (4.2,4.3)	<.0001	4.2 (4.2,4.2)	4.1 (4.1,4.2)	<.0001
Ethnicity (%)				.0204			.0001
Mexican American	5.2 (4.4,6.2)	6.6 (5.5,7.8)	5.4 (3.9,7.5)		3.7 (3.0,4.5)	3.8 (2.6,5.5)	
Other Hispanic	4.3 (3.5,5.2)	4.9 (4.0,5.9)	2.8 (1.5,5.5)		3.7 (2.9,4.7)	4.7 (2.9,7.6)	
Non-Hispanic White	77.3 (75.3,79.3)	74.9 (72.7,77.1)	76.4 (71.1,81.0)		80.6 (78.6,82.5)	73.8 (68.9,78.2)	
Non-Hispanic Black	8.1 (7.1,9.2)	7.7 (6.8,8.8)	11.1 (8.6,14.3)		7.8 (6.7,9.1)	14.1 (11.0,17.8)	
Other race	5.1 (4.5,5.7)	5.9 (5.2,6.8)	4.2 (2.4,7.4)		4.3 (3.6,5.0)	3.6 (1.9,6.6)	
Education (%)				<.0001			<.0001
<9th grade	4.5 (4.1,5.0)	5.2 (4.7,5.8)	8.7 (6.3,11.9)		3.1 (2.5,3.7)	7.5 (5.5,10.2)	
9 to 11th grade	12.6 (11.6,13.7)	12.5 (11.3,13.9)	17.4 (13.7,21.8)		11.9 (10.8,13.1)	18.2 (14.6,22.4)	
High school or equivalent	25.7 (24.5,26.9)	26.2 (24.7,27.8)	28.7 (23.6,34.4)		24.4 (22.9,25.8)	31.6 (25.8,38.0)	
Some college or AA degree	33.8 (32.3,35.2)	31.1 (29.4,32.9)	32.6 (27.0,38.7)		37.2 (35.4,39.0)	32.3 (26.8,38.4)	
College graduate or above	23.4 (21.7,25.2)	24.9 (22.9,26.9)	12.6 (8.7,17.9)		23.5 (21.3,25.8)	10.3 (6.9,15.2)	
Smoke (%)				.1621			.0399
Every day	33.8 (32.4,35.3)	31.7 (30.2,33.3)	31.5 (25.9,37.7)		35.8 (33.7,38.1)	43.5 (37.6,49.6)	
Some days	7.4 (6.8,8.0)	8.1 (7.3,9.1)	5.1 (3.2,8.1)		6.8 (6.1,7.6)	5.5 (3.3,9.1)	
Not at all	58.8 (57.2,60.3)	60.1 (58.4,61.8)	63.4 (56.8,69.4)		57.4 (55.1,59.5)	51.0 (44.7,57.2)	
Alcohol (%)				.0001			<.0001
Yes	86.7 (85.6,87.7)	92.0 (91.1,92.7)	85.4 (80.5,89.2)		81.6 (79.7,83.3)	70.7 (64.9,75.9)	
No	13.3 (12.3,14.4)	8.0 (7.3,8.9)	14.6 (10.8,19.5)		18.4 (16.7,20.3)	29.3 (24.1,35.1)	
Hypertension (%)				<.0001			<.0001
Yes	36.8 (35.5,38.0)	36.7 (35.0,38.5)	52.6 (46.1,59.1)		34.3 (32.7,35.9)	53.7 (47.4,60.0)	
No	63.2 (62.0,64.5)	63.3 (61.5,65.0)	47.4 (40.9,53.9)		65.7 (64.1,67.3)	46.3 (40.0,52.6)	
High cholesterol (%)				.0015			.0185
Yes	41.0 (39.8,42.1)	42.8 (41.2,44.3)	54.5 (47.2,61.6)		37.4 (35.7,39.2)	45.5 (38.6,52.5)	
No	59.0 (57.9,60.2)	57.2 (55.7,58.8)	45.5 (38.4,52.8)		62.6 (60.8,64.3)	54.5 (47.5,61.4)	

Data in the table.

For continuous variables: survey-weighted mean (95% CI), *P*-value was by survey-weighted linear regression (svyglm).

For categorical variables: survey-weighted percentage (95% CI), *P*-value was by survey-weighted Chi-square test (svytable).

ALB = albumin, BMI = body mass index, Incme = ratio of family income to poverty, RA = rheumatoid arthritis.

#### 3.1.2. Relationship between ALB and RA in NHANES

The results of the weighted generalized linear model showed that ALB and RA were strongly associated in each of the different models, and after constant adjustment for confounders, ALB and RA retained this association in the final model 3, with no change in direction and with significance for both males and females. The results are summarized in Table 2. Tables S1 to S3, Supplemental Digital Content, http://links.lww.com/MD/N607 describes the relevant statistics for each model.

**Table 2:** Association between ALB and RA and gender differences, weighted.

	Model 1				Model 2				Model 3			
	Exp (coef)	95% CI low	95% CI upp	*P*-value	Exp (coef)	95% CI low	95% CI upp	*P*-value	Exp (coef)	95% CI low	95% CI upp	*P*-value
Albumin	0.389	0.306	0.493	<.001	0.590	0.457	0.763	<.001	0.662	0.508	0.863	.003
Male	0.358	0.249	0.514	<.001	0.640	0.436	0.940	.025	0.674	0.459	0.989	.046
Female	0.446	0.321	0.619	.000	0.562	0.385	0.823	.004	0.660	0.437	0.998	.049

Model 1 adjust for: none.

Model 2 adjust for: gender, age, ethnicity, education, income.

Model 3 adjust for: gender, age, ethnicity, education, income, smoke, alcohol, BMI, hypertension, high cholesterol, blood glucose.

Exp(coef), HR-hazard ratio; 95% CI, 95% confidence interval.

#### 3.1.3. Results of smoothed curve fitting and threshold effect between ALB and RA in NHANES

**Figure 2.**
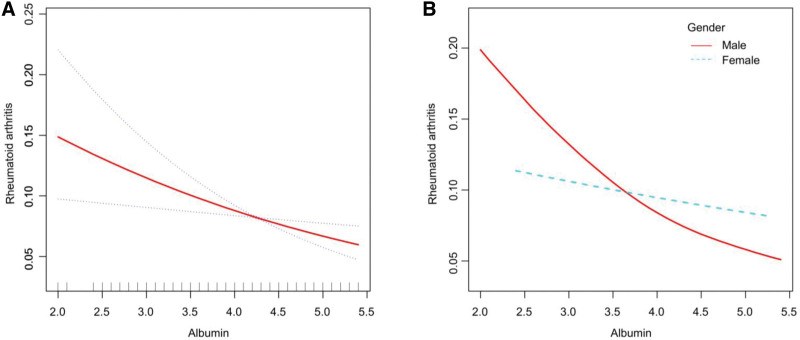
Results of smoothing curve fitting between Albumin and Rheumatoid arthritis.

The generalized additive model was used to test for linear or nonlinear correlations between ALB and RA, and the smooth-fit curve fit of the generalized additive model appropriately depicted the relationship between ALB and RA and maintained the same negative phase relationship as the generalized linear model, and the results are shown in Figure 2(A) and (B). The final 3 models controlling for all covariates were subjected to threshold effect analysis, and the log-likelihood ratio test of *P* > .05 suggested that there was no significant threshold, and the results are shown in Table 3; therefore, there was no significant nonlinear relationship between ALB and RA, and the generalized linear model better depicted the relationship between ALB and RA.

**Table 3:** Results of threshold analysis of the relationship between ALB and RA.

GENDR	Male	Female	Total
Turning point(K)	4.5	4.4	4.4
<K effect	0.5 (0.4, 0.7) <.001	1.0 (0.7, 1.5) .935	0.7 (0.5, 0.9) .010
>K effect	1.4 (0.5, 3.8) .507	0.8 (0.2, 2.6) .677	0.9 (0.5, 1.8) .778
LRT test	0.101	0.731	0.534

Results in table: OR (95% CI) *P*-value.

LRT, log-likelihood ratio test.

### 3.2. MR results

#### 3.2.1. Causal Relationship between ALB and RA

**Figure 3.**
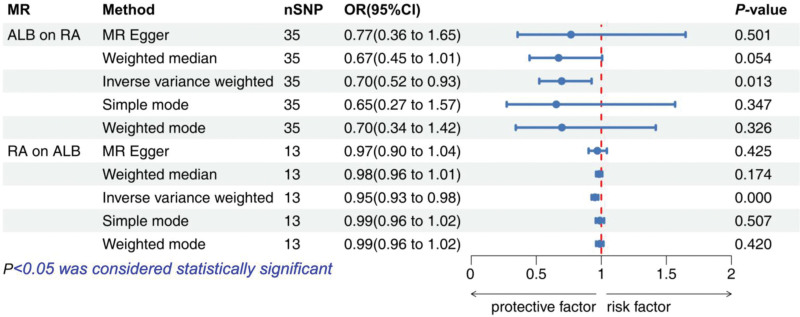
Mendelian Randomization results. ALB, albumin; RA, rheumatoid arthritis; ALB on RA, causality analysis of exposure to albumin on outcome rheumatoid arthritis; RA on ALB, causality analysis of exposure to rheumatoid arthritis on outcome albumin; nSNP, number of single nucleotide polymorphisms; 95% CI, 95% confidence interval.

For MR analysis, we further validated the relationship between ALB and RA by 2-way Mendelian randomization. Through rigorous screening of SNPs and removal of SNPs associated with confounders, 35 SNPs were ultimately used in the causal analysis of ALB on RA and 13 SNPs were used in the causal analysis of RA on ALB. The results of the 5 MR analyses were finally summarized in Figure 3, and the results of the IVW method study showed that there was a bidirectional causal relationship between serum ALB and RA, (IVW: ALB on RA, OR = 0.70 [0.52–0.93], *P* = .013; RA on ALB, OR = 0.95 [0.93–0.98], *P* < .001).

#### 3.2.2. Tests for heterogeneity and horizontal multiple validity

**Figure 4.**
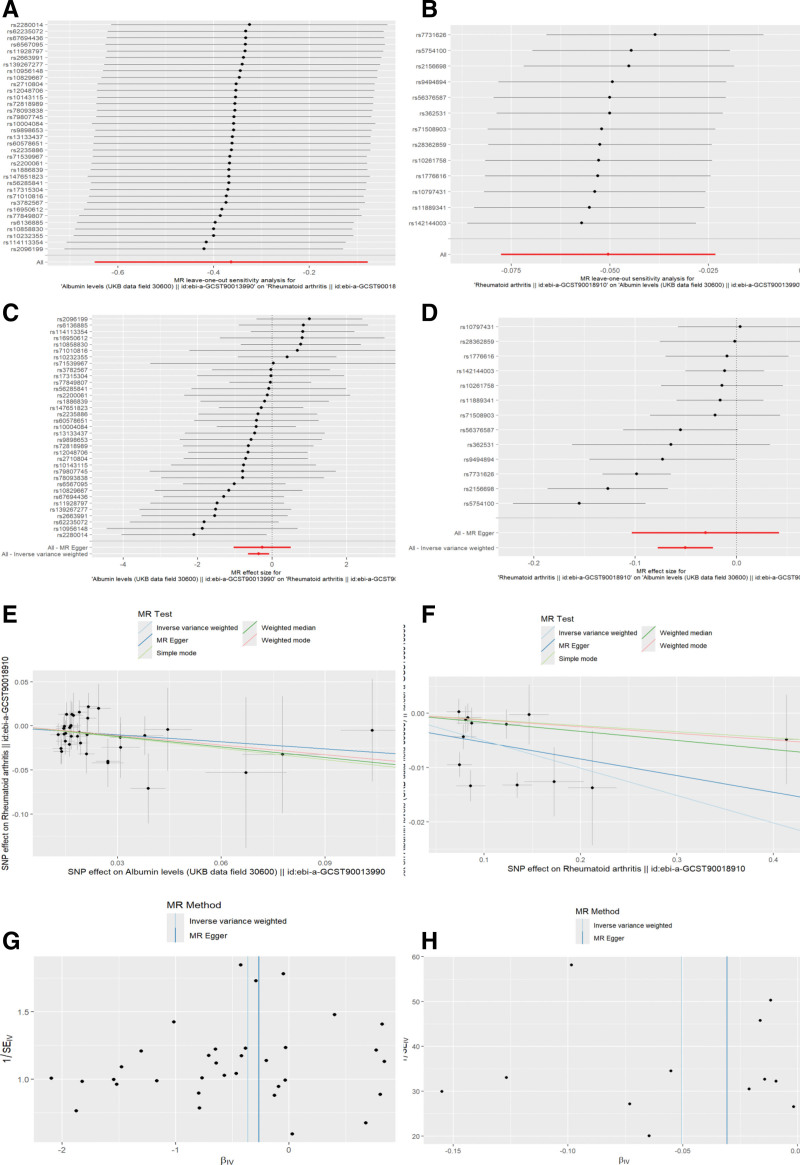
Visualization of heterogeneity and horizontal pleiotropy: leave-one-out method, SNP forest plot, scatterplot, funnel plots.

Accordingly, we further tested the MR results of serum ALB and RA for heterogeneity and horizontal multiple validity, which showed that the causal relationship of ALB to RA was very robust without heterogeneity and horizontal validity, and the causal relationship of RA to ALB by the MR-PRESSO method suggested that there was heterogeneity, but the results were corrected to show that a significant negative correlation still existed (Table 4). plotted leave-one-out, SNP forest plot, scatterplot, and funnel plot of ALB versus RA (Fig. 4: ALB-RA: a, c, e, g; RA-RA: b, d, f, h).

**Table 4:** Heterogeneity and horizontal pleiotropy.

Traits	*F* statistics	MR-PRESSO		Cochran *Q* test (MR-Egger)		Cochran *Q* test (IVW)		Horizontal pleiotropy tests		
		RSSobs	*P*-value	*Q*	*P*-value	*Q*	*P*-value	Intercept	SE	*P*-value
ALB on RA	49.656	37.356	.715	27.818	.723	27.890	.761	−0.002	0.008	.790
RA on ALB	68.469	48.133	<.001	38.184	.000	39.348	.000	−0.002	0.004	.574

ALB on RA = causality analysis of exposure to ALB on outcome RA, RA on ALB = causality analysis of exposure to RA on outcome ALB.

#### 3.2.3. Bayesian co-localization results

**Figure 5.**
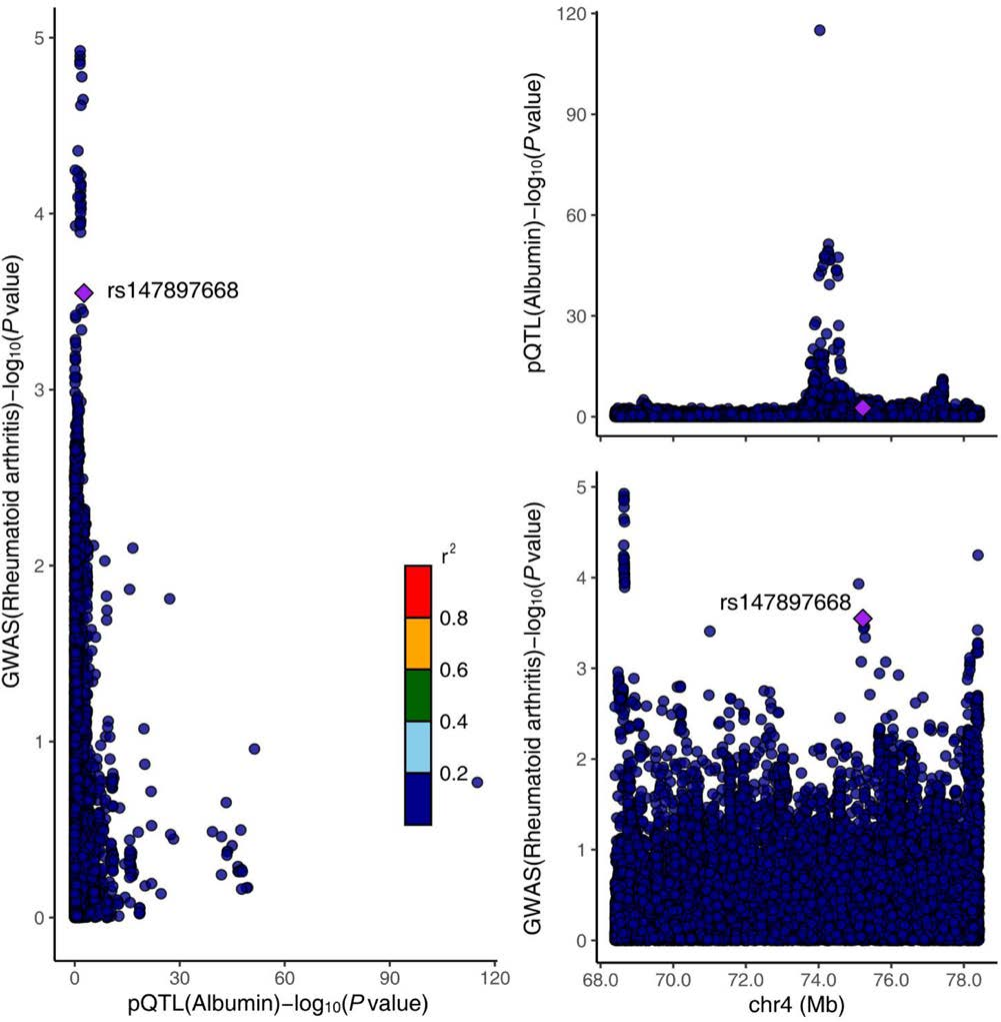
Relationship between genetic variation on Albumin and Rheumatoid arthritis, Using the Rheumatoid arthritis dataset and the 1 Mb base region of Albumin levels in blood. pQTL, protein quantitative trait loci; GWAS, genome-wide association study.

Through Bayesian co-localization analysis, we concluded that PH4 < 0.8, which suggests that this indicates that there is insufficient evidence to support that albumin shares a common genetic locus with RA, and we have visualized the final results of the co-localization analysis as Figure 5

## 4. Discussion

RA is one of the most common immune-mediated inflammatory diseases that, if not recognized and treated in time, can lead to severe joint damage and disability.^[31]^ Despite the progress made in this disease, the diagnosis and treatment of early RA still face great challenges.^[32]^ The pathogenesis of RA, especially in the autoantibody-negative subgroup, is still poorly understood.^[5]^ In seronegative RA patients, the use of other biomarkers can prevent false negatives and aid in the accurate diagnosis of RA.^[33]^ in which ALB was strongly associated with RA and fluctuated in response to changes in RA.^[34]^ However, there are fewer studies between ALB and RA, lacking large sample sizes and reliable validation of results.

The analysis of the NHANES study showed a significant negative association between ALB and RA in the unadjusted generalized linear model 1. And the same trend and significance of association existed across gender. This trend of significance and association remained after further addition of the covariant variable, and finally, in model 3 where all variables were added, the 2 subgroups showed that the association remained. No significant thresholds were found in the smoothed curves based on the generalized summation model, suggesting that ALB and RA are linearly related and that a generalized linear model is more appropriately fitted. In further MR analysis, the IVW method in bidirectional MR showed a negative and significant association between ALB and RA in both directions. The direction of the association remained consistent in the remaining 4 methods. There was no significant heterogeneity or horizontal pleiotropy detection in the causal relationship of ALB to RA, and the results of the leave-one-out method remained relatively robust regardless of which SNP result was excluded, and the overall range of variation of the error line was small. Meanwhile, the SNP forest plot showed that most of the SNP results were negatively correlated. The scatterplot showed that all MR methods had consistent directionality the Egger intercept was almost zero, and the funnel plot was roughly symmetrical, so ALB reduction was a risk factor for RA and the results were very robust. In the causal analysis of RA on ALB, the IVW method suggested that it was also negatively correlated, and the results of the remaining 4 methods were similar. Cochran *Q* test, funnel plot, and MR-PRESSO method suggested the presence of heterogeneity, but the corrected results showed that there was still a significant negative correlation, which indicated that although there was heterogeneity among the selected instrumental variables, it did not affect the results of IVW. Therefore the results remain robust. Accordingly, we believe that the results of NHANES and MR analyses doubly demonstrate the existence of a negative correlation between ALB and RA. Unfortunately, however, in the Bayesian co-localization analysis, we did not find sufficient evidence to support that they share common genetic loci, a result that suggests that there may not be a direct genetic link between ALB and RA and that they may affect patients through different biological pathways.

The bidirectional causality between RA and ALB may be due to the fact that on the 1 hand, nutritional deficiencies and decreased immunity caused by hypoproteinaemia may contribute to the onset of RA, and on the other hand, RA can likewise cause a decrease in ALB, thus appearing to be a bidirectional causality. The negative correlation between ALB and RA may be caused by these mechanisms. Firstly, with the gradual progression of RA, about 24.7% of patients with RA develop malnutrition with hypokalemia.^[35]^ The lowering of ALB may reflect the deterioration associated with RA.^[36]^ This characteristic progression of malnutrition in RA patients is associated with advanced age, increased inflammatory disease activity, and decreased quality of life.^[37]^ Wasting atrophy attributable to excessive proteolytic metabolism and useless atrophy caused by inflammatory cytokines,^[35]^ namely tumor necrosis factor-alpha (TNFα), interleukin 1 (IL-1), and interferon-gamma, which are mediators of RA inflammation,^[38]^ that regulate fat, carbohydrate and protein metabolism.^[39]^ Hypermetabolism driven by these cytokines can cause malnutrition in hypoALBemia, and, in addition, dysfunction leads to wasting muscle atrophy, which further accelerates protein catabolism. Second, ALB exchange between plasma and synovial fluid exists in RA and is in dynamic equilibrium.^[40]^ Synovial permeability is increased in patients with RA, e.g., 6-fold ALB permeability and approximately 40-fold increase in giant GLB in the knee.^[41]^ Labeling of ALB by radioactive technetium-99m labeling revealed that RA disease synovitis showed traces of ALB in arthrography,^[42]^ demonstrating the transfer of serum ALB into the joints. In addition, ALB clearance in rheumatoid knee arthritis effusions was significantly higher than in knee osteoarthritis effusions.^[43]^ Thus there is a significant metabolic depletion of ALB in the joints, which causes intra-articular transfer of serum ALB, further exacerbating the depletion and reduction of ALB. Third, as RA shifts from stable to active phase, urinary ALB excretion increases in RA patients^[44]^ and urinary ALB excretion was significantly correlated with CRP and disease activity in RA patients.^[45]^

The mechanism of the negative association of ALB in RA, i.e., metabolic depletion in synovial fluid and increased synovial permeability, and the transport of serum ALB into the joints, thus making ALB inflammatory-targeting ability, and the ability of ALB to actively target disease sites in RA, along with leukocyte’s excellent cytocompatibility, degradability in biological tissues, non-antigenicity, and safety profile, implies that ALB can serve as drug carriers for RA therapy.^[46]^ For example, Andreas Wunder et al^[47]^ used an arthritic mouse model to study the pharmacokinetics and efficacy of covalent coupling of methotrexate to ALB and found that the amount of ALB accumulated in inflamed paws was significantly higher, whereas the liver and kidneys contained significantly lower amounts of ALB, which provides additional ideas for the treatment of RA.

Although we double-validated our results, there are some limitations; in NHANES, the pathogenesis and influencing factors of RA patients have not been fully clarified, and there is the possibility of residual confounding, while ALB was affected by a variety of factors, and there may be inaccuracy of measurement. In addition, in MR, different sexes genders and age may have different causal effects, thus failing to stratify the analysis, moreover, there exists developmental compensation (canalization), for certain adverse exposures, individuals may develop compensatory mechanisms during long-term development to reduce the impact of adverse genetic factors, which may cause an overestimation of the effect value. Finally, the population investigated by NHANES was a U.S. population, and, limited to data sources, a European population was selected for MR; the consistency of the results for these 2 populations is an issue that needs to be further explored.

## 5. Conclusion

In conclusion, we believe that there is a negative association between ALB and RA, and the reduction of ALB may be one of the risk factors for RA, but it may also be one of the results during the development of RA.ALB can be used as a biomarker for the auxiliary diagnosis of RA. In addition, for the patients with diagnosed RA, an appropriate high-protein diet can be used as a supplement for the depletion of ALB, meanwhile, ALB is expected to be a new drug carrier for RA.

## Acknowledgments

We thank all the volunteers and participants who participated in the National Health and Nutrition Examination Survey and Whole Gene Association Sequencing, and the designers of the R language and R package. We thank them for making this study possible with their free dedication.

## Author contributions

**Conceptualization:** Ke Liu, Le Zhang, Ziming Zhang, Liang Ou, Jianjun Kuang.

**Data curation:** Ke Liu, Le Zhang, Haoming Zhao, Zuyu Tang, Sheng Hua, Yixiao Xiong.

**Formal analysis:** Ke Liu, Le Zhang, Haoming Zhao.

**Methodology:** Ke Liu, Le Zhang, Zuyu Tang.

**Validation:** Sheng Hua, Yixiao Xiong.

**Visualization:** Sheng Hua, Yixiao Xiong, Ziming Zhang.

**Writing – original draft:** Ke Liu, Le Zhang, Liang Ou, Jianjun Kuang.

**Writing – review & editing:** Ke Liu, Le Zhang, Liang Ou, Jianjun Kuang.

## Supplementary Material


