# Supplementary material for: Relationship between albumin and rheumatoid arthritis: Evidence from NHANES and Mendelian randomization
Source: Medicine (Baltimore). 2024 Oct 11;103(41):e39776. doi: 10.1097/MD.0000000000039776 (PMC11479416; doi:10.1097/MD.0000000000039776)
Supplement: Supplementary file 1 [file medi-103-e39776-s001.docx]

**Relationship between Albumin and Rheumatoid arthritis: evidence from NHANES and Mendelian randomization**

**Ke Liu: First author, Hunan University of Chinese Medicine, Changsha 410208, Hunan, China**

**Supplementary Table S1. Correlation statistics for generalized linear model 1**

|  | Male | Female | Total |
| --- | --- | --- | --- |
| AIC: | 2880.759 | 2382.88 | 5290.077 |
| Log Likelihood: | -1523.9539 , df= 3 | -1289.6035 , df= 3 | -2833.4696 , df= 3 |
| Null.deviance | 3089.9671 on 6985 degrees of freedom | 2603.8153 on 4992 degrees of freedom | 5741.2246 on 11978 degrees of freedom |
| deviance | 3047.9077 on 137 degrees of freedom | 2579.2071 on 137 degrees of freedom | 5666.9391 on 137 degrees of freedom |
| residuals SD | 4.815 (pearson chi-square normality test *P*= <0.0001 ) | 4.369 (pearson chi-square normality test *P*= <0.0001 ) | 4.5996 (pearson chi-square normality test *P*= <0.0001 ) |
| Number of observations used: | 6986 | 4993 | 11979 |

AIC, Akaike Information Criterion; df, degrees of freedom.

**Relationship between Albumin and Rheumatoid arthritis: evidence from NHANES and Mendelian randomization**

**Ke Liu: First author, Hunan University of Chinese Medicine, Changsha 410208, Hunan, China**

**Supplementary Table S2. Correlation statistics for generalized linear model 2**

|  | Male | Female | Total |
| --- | --- | --- | --- |
| AIC: | 2781.0916 | 2291.7716 | 5064.7538 |
| Log Likelihood: | -1453.7216 , df= 13 | -1212.9085 , df= 13 | -2688.3316 , df= 14 |
| Null.deviance | 3089.9671 on 6985 degrees of freedom | 2603.8153 on 4992 degrees of freedom | 5741.2246 on 11978 degrees of freedom |
| deviance | 2907.4432 on 127 degrees of freedom | 2425.8171 on 127 degrees of freedom | 5376.6632 on 126 degrees of freedom |
| residuals SD | 4.8318 (pearson chi-square normality test *P*= <0.0001 ) | 4.2049 (pearson chi-square normality test *P*= <0.0001 ) | 4.5374 (pearson chi-square normality test *P*= <0.0001 ) |
| Number of observations used: | 6986 | 4993 | 11979 |

AIC, Akaike Information Criterion; df, degrees of freedom.

**Relationship between Albumin and Rheumatoid arthritis: evidence from NHANES and Mendelian randomization**

**Ke Liu: First author, Hunan University of Chinese Medicine, Changsha 410208, Hunan, China**

**Supplementary Table S3. Correlation statistics for generalized linear model 3**

|  | Male | Female | Total |
| --- | --- | --- | --- |
| AIC: | 2770.9273 | 2274.5484 | 5031.6422 |
| Log Likelihood: | -1443.2996 , df= 20 | -1198.6058 , df= 20 | -2665.8537 , df= 21 |
| Null.deviance | 3089.9671 on 6985 degrees of freedom | 2603.8153 on 4992 degrees of freedom | 5741.2246 on 11978 degrees of freedom |
| deviance | 2886.5991 on 120 degrees of freedom | 2397.2116 on 120 degrees of freedom | 5331.7074 on 119 degrees of freedom |
| residuals SD | 4.9276 (pearson chi-square normality test *P*= <0.0001 ) | 4.5782 (pearson chi-square normality test *P*= <0.0001 ) | 4.6526 (pearson chi-square normality test *P*= <0.0001 ) |
| Number of observations used: | 6986 | 4993 | 11979 |

AIC, Akaike Information Criterion; df, degrees of freedom.
